# Neurodevelopmental Disorder and Cortical Myoclonus in *ZMYM2* Deficiency

**DOI:** 10.1002/mds.70056

**Published:** 2025-09-24

**Authors:** Luca Pollini, Maria Novelli, Lorena Travaglini, Fabiola Panvino, Vincenzo Leuzzi, Francesco Pisani, Serena Galosi

**Affiliations:** ^1^ Department of Human Neuroscience Sapienza University of Rome Rome Italy; ^2^ Laboratory of Medical Genetics, Translational Cytogenomics Research Unit Bambino Gesù Children's Hospital, IRCCS Rome Italy

*ZMYM2* encodes a regulatory protein that functions as a transcriptional corepressor by interacting with several nuclear receptors. *ZMYM2* autosomal dominant pathogenic variants have been associated with a neurodevelopmental‐craniofacial syndrome with variable renal and cardiac abnormalities.^1^, ^2^

In this letter, we describe the first case of *ZMYM2* deficiency associated with prominent neurological phenotype and cortical myoclonus.

Our patient presented with developmental delay and stereotypies, prompting an initial diagnosis of autism spectrum disorder. Since the age of 3 years he suffered from generalized tonic‐clonic seizure and focal motor seizure. A diagnosis of mild intellectual disability was made at the age of 5 years.

He exhibited bilateral hand jerky movements since childhood. At age 14, he came to our attention because of a progressive spastic gait and sensitivity deficits in the lower limbs and abdomen caused by a thoracic spinal lesion from severe kyphoscoliosis, which required surgical intervention.

On examination at age 18, he had an asymmetrical spastic paraplegia and a high‐frequency jerky movement of the upper and lower limbs that were aggravated by outstretching and opening and closing his hands, and on feet dorsiflexion (Video S1). Jerks could be triggered by tactile stimulation of the hands.

**Video 1:** While the patient keeps his arms outstretched, there are frequent and low‐amplitude distal jerks in both hands, which are more evident during movement or when the hands are held outstretched. Occasionally, higher‐amplitude jerks are superimposed on the smaller ones. The frequent jerks significantly diminish when the patient relaxes his hands (segment 1). During foot dorsiflexion, frequent and irregular jerks, with a higher amplitude compared with jerks observed in the hands, are present (segment 2). Segment 3 shows the patient's gait that is characterized by an asymmetrical pattern with mixed spastic and sensory ataxic components.

A neurophysiological examination helped classify its involuntary movement as myoclonus because of the presence of short and arrhythmic electromyographic (EMG) bursts, synchronous between agonist and antagonist muscles. Jerk‐locked back‐averaging, corticomuscular coherence and phase analysis showed the cortical origin of myoclonus (Fig. 1).

**FIG. 1:**
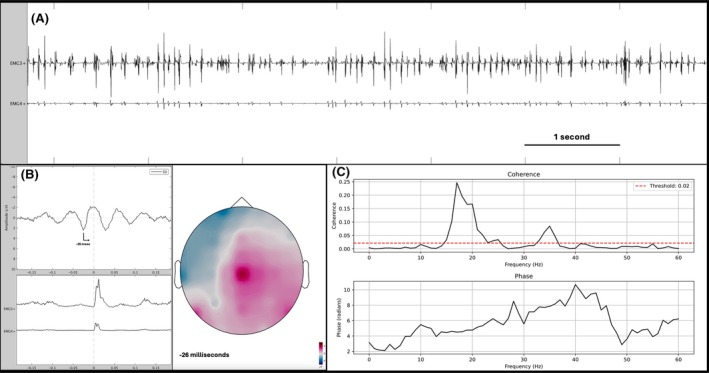
(**A**) Electromyographic (EMG) recording that documents the presence of arrhythmic bursts of short duration (mainly <50 ms), synchronous between tibialis anterior (EMG3+) and gastrocnemius (EMG4+). (**B**) Jerk‐locked back‐averaging of 100 EMG bursts, demonstrating a transient waveform of around 4 μV at the Cz electrode with a maximal positive peak occurring 26 ms before the rectified EMG bursts. A voltage map at −26 ms from the EMG bursts shows the maximal positive polarity (in red) at Cz electrode. The blue area indicates negative voltage areas, and the white area an isoelectric signal. (**C**) Corticomuscular coherence (CMC) and phase analysis between Cz and EMG3+ signals. CMC analysis shows the presence of a significant peak, exceeding the 95% significance level threshold (dotted red line), between 15 and 23 Hz and a smaller peak around 35 Hz. Phase analysis shows an ascending slope, indicating that the electroencephalographic signal precedes the EMG signal with a time lag of 27 ms between 15 and 23 Hz. [Color figure can be viewed at wileyonlinelibrary.com]

A whole‐exome sequencing disclosed the presence of a novel de novo likely pathogenic truncating variant c.421C>T; p.Arg141Ter in *ZMYM2*.

The neurological phenotype associated with ZMYM2 deficiency is expanding rapidly, with new cases reported in the literature suggesting links with epilepsy, Rett‐like phenotypes, cerebral palsy mimics, and spastic diplegia.^1^, ^2^, ^3^ Unlike several previously reported cases, this patient showed no renal or cardiac involvement, but rather a predominantly neurological phenotype characterized by epilepsy and cortical myoclonus. Cortical myoclonus has not yet been associated with *ZMYM2* deficiency, further broadening its neurological spectrum.

Cortical myoclonus is defined as a brief and sudden muscular activation arising from cortical areas. Although in many cases the distinction is straightforward, high‐frequency myoclonus can mimic a tremor, and neurophysiological analysis can aid the diagnosis,^4^ as observed in our patient. The distinction is crucial because myoclonus and tremor underpin different pathophysiological mechanisms and require different treatments.

Before the association of *ZMYM2* deficiency with human disease was elucidated, tremor was reported in a boy with a neurodevelopmental disorder. This boy had a 2.1‐Mb deletion on chromosome 13q12.11 that included the *ZMYM2* gene.^5^

However, no further neurophysiological study was performed to elucidate the nature of his movement disorders, and the deletion contained other genes in addition to *ZMYM2*.

In summary, we present a novel phenotype characterized by neurodevelopmental disorder, epilepsy, and movement disorder in *ZMYM2* deficiency, suggesting that cortical myoclonus may be part of the *ZMYM2*‐related neurological spectrum.

Further characterization of additional patients is required to definitively include *ZMYM2* on the list of genes responsible for myoclonic syndromes associated with neurodevelopmental disorders.

## Ethical Compliance Statement

Written informed consent for offline and online video distribution of the video material was obtained from the patient and his caregiver and is available upon request.

## Author Roles

(1) Research project: A. Conception, B. Organization, C. Execution;

(2) Data Acquisition and Analysis: A. Acquisition, B. Analysis;

(3) Manuscript Preparation: A. Writing of the first draft, B. Review and Critique.

L.P.: 1A, 1B, 1C, 2A, 2B, 3A, 3B.

M.N.: 1B, 1C, 2A, 3B.

L.T.: 2B, 3B.

F. Panvino: 2B, 3B.

V.L.: 2B, 3B.

F. Pisani: 2B, 3B.

S.G.: 1A, 1B, 1C, 3A, 3B.

## Supporting information


**Data S1.** Supporting Information

## Data Availability

The data that support the findings of this study are available on request from the corresponding author. The data are not publicly available due to privacy or ethical restrictions.
