# Supplementary material for: Neurodevelopmental Disorder and Cortical Myoclonus in ZMYM2 Deficiency
Source: Mov Disord. 2025 Sep 24;40(12):2839–41. doi: 10.1002/mds.70056 (PMC13001705; doi:10.1002/mds.70056)
Supplement: Supplementary file 2 — Data S1. Supporting Information [file MDS-40-2839-s001.docx]

**Supplementary Material**

EEG and EMG recording was sampled at at 1024 Hz. EEG and EMG signals were pre-processed using a 1 Hz and 10 Hz high-pass filters and EMG signal was rectified.

For jerk-locked back-averaging (JLBA), markers were manually set at the onset of 100 jerks, according to a visual inspection and equal lenght epochs were created and subsequently averaged.

Signal pre-processing and JLBA was performed using Brainstorm, **[1**], which is documented and freely available for download online under the GNU general public license (<http://neuroimage.usc.edu/brainstorm>).

Cortico-muscular coherence and phase analysis was computed using a custom-made software. For coherence analysis data were segmented in 1-sec epochs with a 50% overlap window. The magnitude squared coherence was computed and the confidence limit was set at 95% [2].

Phase analysis was calculated on the same segments and estimated conduction time was calculated according to previously described methods [3].

Supplementary References

1. Tadel F, Baillet S, Mosher JC, Pantazis D, Leahy RM. Brainstorm: a user-friendly application for MEG/EEG analysis. *Comput Intell Neurosci*. 2011;2011:879716. doi:10.1155/2011/879716
2. Halliday, D.M., Rosenberg, J.R., Amjad, A.M., Breeze, P., Conway, B.A., Farmer, S.F., 1995. A framework for the analysis of mixed time series/point process data—Theory and application to the study of physiological tremor, single motor unit discharges and electromyograms. Prog. Biophys. Mol. Biol. 64, 237–278. <https://doi.org/10.1016/S0079-6107(96)00009-0>
3. Grosse P, Guerrini R, Parmeggiani L, Bonanni P, Pogosyan A, Brown P. Abnormal corticomuscular and intermuscular coupling in high-frequency rhythmic myoclonus. *Brain*. 2003;126(Pt 2):326-342. doi:10.1093/brain/awg04
